# Measuring Salivary Cortisol to Assess the Effect of Natural Environments on Stress Level in Acute Patients With Severe Brain Injuries: An Exploratory Study

**DOI:** 10.7759/cureus.44878

**Published:** 2023-09-07

**Authors:** Jane Jöhr, Tania Martinez, Renaud Marquis, Stephen Bruce, Pierre-Alain Binz, Sabine Rey, Gaël Hafner, Caroline Attwell, Karin Diserens

**Affiliations:** 1 Department of Clinical Neurosciences/Neurology, Lausanne University Hospital and University of Lausanne (CHUV-UNIL), Lausanne, CHE; 2 Laboratory of Clinical Chemistry/Biomedicine, Lausanne University Hospital and University of Lausanne (CHUV-UNIL), Lausanne, CHE

**Keywords:** nature, stress, neurorehabilitation, brain injury, acute phase, salivary cortisol

## Abstract

Background: Salivary cortisol is a safe and non-invasive measure of hypothalamic-pituitary-adrenal axis function and is used as a biomarker of the human stress response. Natural environments are recognized to contribute to help reduce the effect of stress.

Objective: To determine the feasibility of a salivary cortisol collection protocol for acute severely brain-injured patients, and to explore the influence of exposure to natural settings on salivary cortisol concentration as an index of stress level.

Methods: An exploratory study on 17 acute patients with severe brain injury was performed. We collected salivary samples in a closed hospital ward and a therapeutic garden at the start of the session and after 30 minutes of rest time. Physiological parameters, level of communication, and subjective well-being were also assessed.

Results: The primary objectives regarding the feasibility of the protocol were met overall. We found no significant differences in cortisol values when including the whole population. However, cortisol values were significantly higher in the indoor environment in patients with communication attempts.

Conclusions: A salivary collection protocol with brain-injured patients in the acute phase is feasible and safe, and this type of measurement could pave the way for future research supporting the benefits of nature as an additional resource in their neurorehabilitation.

## Introduction

In Switzerland, more than 130,000 people suffer from brain injuries due to a variety of causes [1]. Every year, almost 22'000 people develop a cerebral lesion of which about 16'000 are diagnosed with a stroke [2]. The diversity of etiologies leading to acquired cerebral damage implies that any person could potentially be affected during their lifetime, independently of age, sex, or health status. As this is a highly stressful event with significant repercussions on different levels (individual, social, and public health), it is important to have efficient resources to improve the recovery and prognosis of these patients.

Nature is known to have a positive influence on health and to mitigate the negative effects of stress. Exposure to nature through access to an outdoor environment, such as a garden, may provide a complementary approach to early neurorehabilitation for brain-damaged patients [3]. Indeed, exposure of the general population to green spaces is beneficial in reducing diastolic blood pressure, heart rate, salivary cortisol levels, and cardiovascular mortality. Further, Ulrich's pioneering study in a hospital setting showed that patients recovered faster after surgery when they viewed trees from their windows rather than empty walls [4]. In the case of head injury patients, nature-based therapies have shown definite benefits. Studies using horticultural therapy reported significant improvement in motor and cognitive function [5,6]. Another study of chronic post-stroke patients showed a significant decrease in anxiety for the group receiving a forest therapy program, compared to the group receiving therapy in an urban area [7]. Finally, our group demonstrated the beneficial impact of an outdoor neurosensory intervention for improving adaptive goal-oriented behavior in the early phase of recovery in patients with limited motor interaction and communication [8].

The physiological response to stress involves the secretion of cortisol, a glucocorticoid hormone in humans that reflects the adaptation of the hypothalamic-pituitary-adrenal (HPA) axis to stressful elements [9]. In blood, cortisol levels peak 15 to 30 minutes after the stressful event and then slowly decline to pre-stress levels 60 to 90 minutes later. Because of the high diffusivity of cortisol into saliva, a constant relationship is maintained between blood and saliva levels. Therefore, the measurement of cortisol levels in saliva represents a reliable, repeatable, secure, and non-invasive means of detecting stress in a particularly vulnerable population [10].

The effects of cortisol in relation to brain injury are still unclear. According to a recent systematic scoping review, some studies show a decrease in cortisol secretion after traumatic brain injury (TBI), while others show no difference in the HPA axis response [11]. Furthermore, there does not appear to be a clear correlation between alterations in cortisol levels after TBI and an unfavorable clinical outcome [12]. On the other hand, a significant association is demonstrated between unfavorable psychosocial outcomes and the gradual amendment of impaired self-awareness (i.e., the reduced ability to recognize the deficits caused by neurological damage, common in TBI patients), leading to greater anxiety and stress. In the case of stroke, a systematic review showed a significant correlation between elevated cortisol levels and severe outcome stroke (dependency, morbidity, and increased mortality) [13]. In addition, a meta-analysis concluded that elevated cortisol levels should be considered a biomarker for post-stroke depression [14]. In this context, treatments to lower cortisol could be potential restorative targets to reduce the risk of depression and support emotional adjustment in brain-damaged patients.

To our knowledge, the feasibility of an experimental protocol to collect salivary cortisol as an indicator of stress levels in acute hospitalized brain-injured patients has not yet been investigated. Therefore, in the present study, we explored the feasibility of such a protocol to determine the relevant parameters to be monitored in subsequent larger-scale studies with acute brain-injured patients that could assess the effect of exposure to natural environments on stress levels. We would expect a decrease in salivary cortisol in outdoor environments, indicating the positive effect of natural environments on stress levels. Such an effect would be useful in supporting the therapeutic benefits of nature as an important resource for early neurorehabilitation.

## Materials and methods

Patients

All adult patients with severe brain injury admitted to the Department of Clinical Neurosciences at Lausanne University Hospital between June and November 2020 were consecutively recruited for this exploratory, single-center, prospective, cross-over, randomized study. For study inclusion, participants needed (1) to be at least 18 years of age, (2) to have a confirmed diagnosis of acute severe brain injury, (3) to be hemodynamically and cerebrovascularly stable, (4) to be suitable for intra-hospital transport, and (5) to give their consent to participate in the study (or through their legal representative). Exclusion criteria were chronic illness, progressive neurological disease, pre-existing endocrine dysfunction including adrenal or pituitary insufficiency, tumor of the adrenal glands, any oral cavity disorders that may affect salivary sample collection and psychiatric history.

Outcome measures

The primary outcome was to determine the feasibility of the saliva collection protocol, and we used the following acceptability indicators: (a) inclusion rate (i.e. patients included after initial screening); (b) participation rate (i.e. patients participating in the two scheduled sessions); (c) completion rate (i.e. patients successfully completing both sessions including all clinical data collection and salivary samples performed); (d) canceled, postponed or interrupted session rate (i.e. due to problems with equipment availability, lack of personnel, unavailability of the laboratory, priority medical examination or minor or serious incidents during the sessions).

To determine the feasibility of the saliva analysis, we evaluated the following indicators: (a) collection rate (i.e. number of salivary samples successfully collected); (b) measurability rate (i.e. number of samples with a usable, homogenous, and high enough level of salivary cortisol; (c) reliability rate (i.e. salivary samples of sufficient volume for the laboratory to determine a reliable cortisol measure). For the feasibility of implementing the procedure, we used the following indicators: (a) the number of patients who did not experience pain before or after the sessions based on evaluation scales and (b) a qualitative evaluation by means of a satisfaction questionnaire to be completed by the caregivers monitoring the sessions comprising 12 items (rated on a Likert scale from 1 "strongly agree" to 5 "strongly disagree" and one question to prompt a general comment).

For the secondary outcome, we assessed the effect of the environment in which the session took place on cortisol levels based on the following indicators: (a) the difference in cortisol levels between T0 and T30 (independent of the environment); (b) the difference in cortisol levels between the outdoor and indoor environments; (c) the analysis of variance explained by the covariates.

Procedure

**Figure 1:**
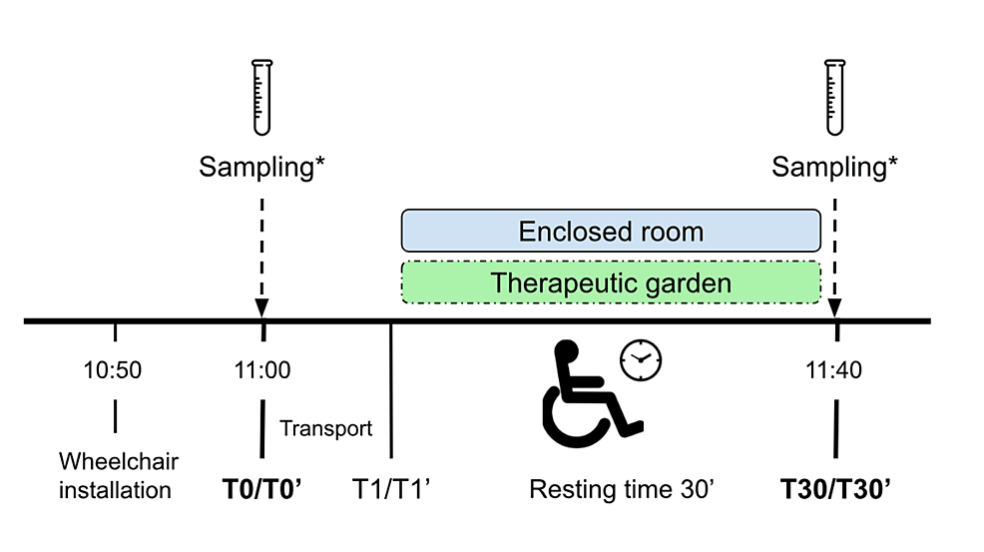
Study protocol. *Sampling includes saliva collection, measurement of physiological and control parameters, report on test conditions, and pain assessment.

Each patient followed a similar baseline protocol conducted over two consecutive mornings and at the same time (11:00 a.m.), to minimize circadian cortisol variations. The protocol consisted of a three-phase session taking place in one of two environments chosen randomly: an indoor environment (T0-T1-T30) or an outdoor environment (T0'-T1'-T30') (Figure 1). Patients were randomly assigned to the order of the conditions (i.e., indoor condition on the first day and outdoor condition on the next day; outdoor condition on the first day and indoor condition on the next day). The average duration of each three-phase session was 45 minutes, with 30 minutes of rest and 15 minutes of transport and data collection. Each session was conducted in the presence of two caregivers (including a physiotherapist, neuropsychologist, or senior medical student).

Study protocol in a three-phase session

T0/T0': Once the patient was installed in the wheelchair, we gave a simple verbal explanation to the patient regarding the conduct of the session. Then, we recorded physiological parameters, control data, and pain assessment (see below for pain assessment procedure) in a pre-defined evaluation grid. After that, the care provider on the ward performed the T0/T0' saliva collection.

Between T0/T0' and T1/T1': We transported the patient to either a closed room or a therapeutic garden according to the randomization. We performed all transport with an elevator passage to replicate the same journey in both situations.

T1/T1': We gave verbal instruction to the patient to keep comfortably still and relax for 30 minutes. We gave no other external stimuli during this period. The caregivers remained in the patient's field of view and only interacted with the patient if they felt it was necessary to repeat the instruction to rest or to secure the patient. However, caregivers kept a sufficient distance and limited direct interaction to maintain a calm and passive attitude.

T30/T30': After 30 minutes, we again recorded physiological parameters, control data, and pain assessment in the assessment grid and took the second saliva sample. We also reported on the test conditions (i.e. any interruptions, noises, problems that occurred).

Experimental environments

Indoor environment: an enclosed room without windows located on the same floor as the inpatient included in the study. For each indoor session, we installed the patient so that the caregivers and the exit door were in the patient’s field of vision throughout.

Outdoor space (therapeutic garden): a garden of about 300 m^2^ located within the hospital grounds, used only by patients, and specifically designed to be safe and allow easy access to patients with reduced mobility. The garden design aims to increase multi-sensory stimulation by offering various structures stimulating the senses (a variety of plants and trees, aromatic plants, and a fountain). For each garden session, we installed the patient in such a way as to have nature and the caregivers in their field of vision throughout.

Data collection

We collected sociodemographic data, medical diagnoses, and medications from patients’ medical records. We used the Modified Rankin Scale (mRS), a five-level scale to categorize the level of functional independence in relation to activities of daily living (no symptoms, no disability despite symptoms, mild disability, moderate disability, moderately severe disability, severe disability). The Glasgow Coma Scale was used as an indicator of the level of consciousness. We assessed the patient's level of communication using four categories (functional, intentional, communication attempts, and absent).

Saliva cortisol sampling

We collected saliva samples using the Salivette® Cortisol system (Sarstedt AG, Ltd, Nümbrecht, Germany). This system reportedly yields a cortisol collection recovery rate of close to 100%, regardless of analyte concentration and volume [15]. The system has previously been demonstrated to be a practical and accurate technique for passive saliva collection [10]. Due to the possibility of impaired consciousness in the study population subjects, we adapted the saliva collection instructions to optimize the safety of the collection. For this purpose, we tied the absorbent pad provided with surgical thread, allowing medical staff to maintain the device throughout the collection to prevent accidental ingestion of the material. We introduced the Salivette® into the patient's oral cavity with anatomical forceps and moved it around according to the position of the salivary glands (i.e. lower gingivobuccal sulcus, lower gingivolabial sulcus, oral floor). In cases where salivation was low, the care provider invited the patient to perform masticatory movements to stimulate saliva production. The Sarstedt AG recommendations state that the device should be held for a minimum of 120 seconds for collection; we increased this time due to the low amount of saliva in this patient population. We performed the saliva collection in the same way for each participant. Once the samples were collected, they were either sent directly to the in-hospital clinical chemistry laboratory or stored in the department's refrigerator and then brought to the laboratory on the same day.

The collected saliva samples were centrifuged at the clinical chemistry laboratory of the Lausanne University Hospital (CHUV) (ISO 15189:2012 accreditation) at 1000 g for 2 minutes to extract the contents of the absorbent pad, and then the samples were analyzed by tandem mass spectrometry coupled to liquid chromatography (LC-MS/MS). The optimal saliva volume for this LC-MS/MS method is > 1 mL. The minimum saliva volume is 500 μL. Sub-optimal volumes validated by the laboratory (between 500 µL and 1 mL) required a 1:1 dilution (max. 2X) to obtain a measurable volume of 1 mL. Of note, the LC-MS/MS technique is specific for cortisol and, therefore, does not suffer from cross-reactions that can potentially cause interference with other methodologies.

Physiological parameters and external factors

Physiological parameters including heart rate (HR, beats per minute, bpm), systolic (SAP; mmHg), diastolic (DAP; mmHg), and oxygen saturation (SpO2) were recorded simultaneously with the saliva cortisol measure. In addition, we reported certain external conditions of the test, including the day's weather, temperature, humidity, possible noise pollution, and the patient's medication on the day of the testing in the evaluation grid. We also recorded the evaluation of pain at the time of the session (digital scale or Critical-Care Pain Observation Tool, cPOT) in the grid.

Data analysis

We generated descriptive statistics to assess patient characteristics and salivary cortisol levels (i.e., mean, standard deviation, frequency). We described the various indicators of the feasibility as percentages. We considered the protocol feasible if more than 90% of the patients could be included, could participate, and could complete the sessions, if more than 85% of the salivary samples could be adequately collected, and if more than 80% of the samples could be validated for assay.

To explore the effect of the environment on the level of salivary cortisol, we first used a multiple linear regression with the sequential selection of predictor variables and then repeated the procedure to include interactions between environments and predictors. Second, we took the predictors retained by the model with interactions and added a random factor per patient to take into account inter-individual variability and built a multilevel hierarchical model (mixed model). Then, we performed post-hoc tests with correction for multiple comparisons using the FDR (false discovery rate) method. Finally, we used marginal means to assess the directionality of the effects found. We set statistical significance at p < 0.05 and used IBM SPSS Statistics for Windows, Version 26.0 (IBM Corp., Armonk, NY) and R Statistical Software (Version 4.1.2. R Core Team 2020) for analysis, and RStudio (RStudio Team 2020) for graphs.

## Results

Demographics

Seventeen patients were consecutively included (eight males and nine females) and one patient participated in the study twice (two weeks apart), with 36 sessions performed. The average age of the patients was 55 years (22 to 87 years). The mean length of hospitalization was 43 days (range nine to 88 days). Demographics and clinical characteristics are presented in Table 1 and the location of lesions by patient in Table 2.

**Table 1:** Demographics and clinical characteristics. *Note*. Values are provided as *n *(%) unless otherwise indicated.

Demographics (N = 17)	
Age, years (mean [SD])	55.06 (20.23)
Gender, female	9 (52.9)
Average length of stay (mean [SD])	43.12 (24.75)
Diagnosis	
Ischemic stroke	5 (29.4)
Hemorrhagic stroke	5 (29.4)
Traumatic Brain Injury	5 (29.4)
Meningoencephalitis	1 (5.9)
Cerebral anoxia	1 (5.9)
Clinical characteristics at time of assessment (N = 18)	
Modified Rankin Scale	
Moderate disability	1 (5.6)
Moderately severe disability	14 (77.8)
Severe disability	3 (16.7)
Pain assessment	
Slight discomfort	4 (22.2)
Communication	
Functional	5 (27.8)
Intentional	10 (55.6)
Attempts	3 (16.7)
Medication use, yes	
Corticosteroids	3 (16.7)
Melatonin	5 (27.8)
Beta-blockers	12 (66.7)
Neuroleptic or antidepressant	5 (27.8)
Diuretic	1 (5.6)

**Table 2:** Location of lesions and etiology by patient

Patient ID	Etiology	Location of lesions
P1	Traumatic Brain Injury	Multiple intracranial bleeds, in the form of diffuse supra-tentorial subarachnoid hemorrhage, fine subdural hematomas (4 mm), small hemoventriculus in the occipital horns of the lateral ventricles, and possible right temporo-polar contusion.
P2	Hemorrhagic stroke	Fisher IV subarachnoid hemorrhage with signs of hydrocephalus associated with a 3.7x3.4mm intracranial aneurysm of the right carotid terminal.
P3	Cerebral anoxia	Sequelae of severe anoxia affecting bilateral temporal, insular, hippocampal and anterior cingulate cortices and ganglia.
P4	Traumatic Brain Injury	Two diffuse hemorrhagic axonal lesions, one right thalamic and the other mesencephalic. Minimal hemoventriculus within the posterior horn of the right lateral ventricle.
P5	Traumatic Brain Injury	Left frontal subarachnoid hemorrhage with flooding of the homolateral lateral ventricle. Subdural hematoma in the cerebellar tent on the right.
P6	Hemorrhagic stroke	Large intraparenchymal hemorrhage in the posterior fossa centered on the left, associated with filling of the basal cisterns without signs of involvement. Extensive intraventricular hemorrhage without evidence of hydrocephalus.
P7	Hemorrhagic stroke	Fisher IV subarachnoid hemorrhage with cisternal component, on a ruptured 6x5mm aneurysm of the right anterior cerebral artery. Second aneurysm in the right middle cerebral artery with no signs of rupture. Signs of significant intracranial hypertension with the beginnings of falcoral and uncal engagements on the right. Subcutaneous hematoma on the right with convex right subdural hematoma blade.
P8	Ischemic stroke	Partial constitution of homolateral fronto-insular ischemic stroke in the deep and superficial territories of the middle cerebral artery, without hemorrhagic transformation.
P9	Hemorrhagic stroke	Hemorrhagic stroke with hyperacute, acute and subacute component of the deep territory of the left middle cerebral artery, resulting in the formation of a large left temporal hematoma, without underlying arteriovenous malformation or aneurysm.
P10	Ischemic stroke	Acute left pontine ischemic stroke (core: 1.5 mL) and punctiform right posterior parietal subcortical.
P11	Traumatic Brain Injury	Multiple small diffuse linear hyperdensities of subarachnoid locations associated with hemoventriculia of the occipital horn of the right lateral ventricle. Left insular hemorrhagic axonal lesion.
P12	Ischemic stroke	Stroke constituted mainly in the right deep sylvian territory, with a minimal hemorrhagic component at this level as well as in the central sulcus.
P13	Traumatic Brain Injury	Acute left convex subdural hematoma with active bleeding and significant mass effect. Co-associated intra-parenchymal and subarachnoid components.
P14	Hemorrhagic stroke	Ruptured anterior communicating artery aneurysm with panventricular hemorrhagic suffusion (as well as Magendie's and Luschka's foramen), cerebellopontine and optochiasmatic angle cistern, and right sylvian valley, and right frontal communicating intraparenchymal hemorrhage with third ventricle.
P15	Ischemic stroke	Acute ischemic injury with intracerebral hemorrhage type 1 suffusion of the right posterior cerebral territory.
P16	Ischemic stroke	Acute ischemic stroke lenticular, corona radiata and left caudate nucleus body.
P17	Meningoencephalitis	Examination morphologically within the norm for age.

Feasibility outcome measured by indicators

**Figure 2:**
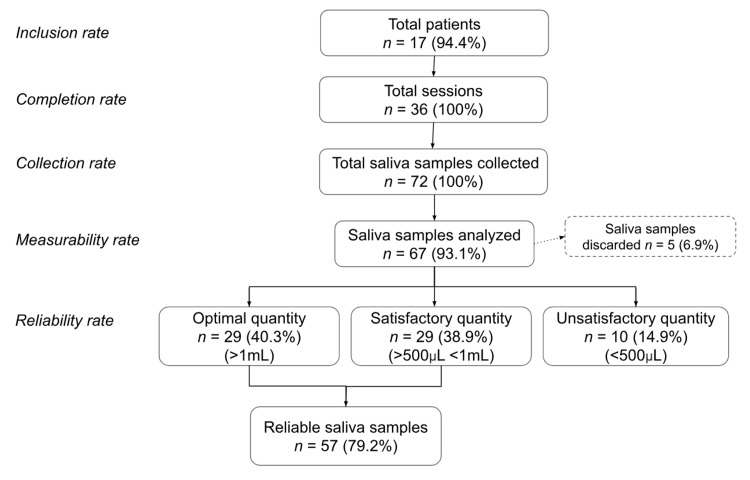
Feasibility outcomes of the saliva collection protocol and saliva analysis.

As shown in Figure 2, 17 patients included in the initial screening consented (or via their relatives) to participate in the study (inclusion rate of 94.4%). All patients successfully participated in both sessions and successfully completed both sessions (participation and completion rate of 100%), resulting in 72 saliva samples (collection rate of 100%). No sessions had to be canceled. One patient had his session moved to the following week due to other medical exams. There were no minor or serious incidents reported during the sessions.

Of the 72 samples collected, 67 could be analyzed by the laboratory (measurability rate of 93.1%). Among these, the laboratory considered 57 as reliable (total reliability rate of 79.2%), including 29 (40.3%) with the optimal volume according to the recommendations (> 1 mL) and 28 (38.9%) with a sub-optimal volume but validated by the laboratory (between 500 µL and 1 mL).

Regarding the feasibility implementation, only two patients reported pain (11.8%), assessed in both as mildly uncomfortable not exceeding 3/10 on the cPOT digital scale. For all patients, the cPOT score never exceeded 2/8. All caregivers (n=5) responded to the satisfaction questionnaire. The results indicated that the collection of clinical data was perfectly adapted to the skill levels of the intervening caregivers. The responders considered the clinical data relevant to the patient's medical condition and were satisfied with the ease with which they could carry out the protocol. Similarly, all agreed that the presence of two care providers per patient was essential, that the length of the session was appropriate, and that patients were comfortable during the saliva collection.

Cortisol measures

In the indoor environment, the mean cortisol levels at baseline (T0) and after 30 minutes (T30) were 9.55 nmol/L and 9.51 nmol/L, respectively. In the outdoor environment, mean values were lower compared to the indoor environment with a mean of 8.01 nmol/L at 'T0' and 8.65 nmol/L at 'T30'. Merging all cortisol values at 'T0' and 'T30', the mean values at 'T0' were 8.67 nmol/L and 9.07 nmol/L at 'T30'.

None of the models applied showed a statistically significant effect regarding cortisol values in relation to either the indoor or the outdoor environment. However, the marginal mean results showed that cortisol levels decrease at 'T30' in both environments (Table 3).

**Table 3:** Marginal means of differences in cortisol levels (T30-T0) from fixed and mixed effects models. Df: Degrees of freedom; Chisq: Chi-squared test

	Value	Df	Chisq	Pr >Chisq
Fixed effects model				
Indoor condition	-2.0177	1	1.3402	0.2665
Outdoor condition	-1.8934	1	1.3258	0.2665
Mixed effects model				
Indoor condition	-1.60219	1	0.7605	0.7227
Outdoor condition	-0.61306	1	0.1259	0.7227

The linear model with interactions (F(15,16) = 1.672; *p *= 0.1592; adjusted R^2^ = 0.25) showed a significant effect of the interaction between the environment and the difference in heart rate (HR) between the two sessions (F(1,16) = 4.52; *p *= 0.049). Post-hoc tests performed showed that the difference in HR was significant in the outdoor environment (F(1,16) = 7.16; *p *= 0.033). The value of the coefficient was negative, which tells us that the higher the HR during the time in the outdoor environment, the lower the cortisol, and vice versa (Table 4).

**Table 4:** Results of the linear mixed effects model (type II Wald Chi-square tests) as a function of the difference in cortisol values between T30 and T0. *p <0.05 mRS: Modified Rankin Scale

	Chisq	Df	*p*-value
Condition	0.914	1	0.339
Sex	1.290	1	0.256
Heart rate T30-T0	0.182	1	0.669
mRS	2.946	1	0.086
Communication	3.364	2	0.186
Condition: Heart rate (T30-T0)	4.046	1	0.044*
Condition: sex	0.519	1	0.471
Condition: communication	6.198	2	0.045*

Regarding the mixed model, the results showed a significant effect of interactions between the environment factor and the level of communication (Wald χ^2^ = 6.198; *p *= 0.045) as well as between the environment factor and the difference in HR between sessions (Wald χ^2^ = 4.046; *p* = 0.044) (marginal R^2^ = 0.19; conditional R^2^ = 0.75; REML (restricted maximum likelihood criterion) at convergence = 145.6) (Table 5). Post-hoc tests for this model showed that the higher the HR, the lower the cortisol, but not in the indoor environment where a lower HR correlated with lower cortisol. In both models, the effect of HR was positive indicating that the HR of the patients was slightly higher at 'T30'.

**Table 5:** Linear model with interactions as a function of the difference in cortisol values between T30 and T0. *p <0.05

	Coefficient	95% CI	*p*-value
Outdoor condition : HR (T30-T0)	-0.84	[-1.69, 0.00]	0.0494*
Post-hoc tests: F Test adjusted slope for HR T30-T0			
Indoor condition	0.123		0.5463
Outdoor condition	-0.431		0.0332*

Regarding the interaction effect between communication level and environment, patients with communication attempts showed significantly higher cortisol levels than patients with intentional communication in the indoor environment (χ^2^ = 7.53, *p *= 0.03646). Evaluating the marginal means, we noted that cortisol values decreased at 'T30' for patients with functional and intentional communication but increased in patients with communication attempts (Table 6).

**Table 6:** Interaction effect between communication level and condition. *p <0.05

	Coefficient	Chisq	*p*-value
Communication attempts – Intentional: indoor	10.652	7.5275	0.0364*
Communication attempts – Functional: indoor	11.287	2.5289	0.3353
Intentional – Functional: indoor	0.635	0.0103	0.9860
Communication attempts – Intentional: outdoor	2.382	0.4326	0.9860
Communication attempts – Functional: outdoor	2.279	0.0932	0.9860
Intentional – Functional: outdoor	-0.103	0.0003	0.9860

## Discussion

In this study exploring the feasibility of a saliva collection protocol to measure salivary cortisol as an index of stress in brain-injured patients during the acute phase, the objectives were met overall. All sessions were successfully realized, we were able to perform all the planned salivary sample collection without incident and we obtained a reasonable number (almost 80%) of samples with a valid saliva volume for assay. All caregivers reported that patients were comfortable during the salivary swabs, most participants did not report any pain during the sessions, and for the minority who did experience pain, they rated it as mild. As the safety and comfort of the patients are key elements, this information provides reassuring arguments regarding the acceptability of the protocol. The results are encouraging and indicate that saliva collection is feasible in this population in the acute phase and allows the detection of salivary cortisol in acute brain-injured patients.

The percentage of invalid samples in our study (6.9%) is comparable to the results of other inpatient studies, including in intensive care [16,17]. Although the intensive care unit (ICU) population is not strictly identical (predominantly intubated), it has some similar characteristics such as reduced oral intake of food and beverages, which negatively influences salivary flow. An alternative hypothesis supporting the hyposialia found in some of our patients is that it relates to medication. Indeed, clinical studies suggest that hyposialia is an almost systematic side effect of patients treated with more than four different drugs simultaneously [18]. In our study, 94.4% of the patients were treated with more than four substances. In addition, some classes of drugs can induce a dry mouth (e.g., diuretics, hypotensive drugs, antipsychotics) [19]. Twelve of our patients (70.6%) were under treatment with one or more of the above-mentioned drugs.

However, the advantages of this protocol are numerous in the acute phase with cerebro-injured patients; it is non-invasive, non-painful, and acceptable even to patients who are incapable of discernment, it does not require the presence of a nursing team and does not need the difficult venous access, which allows the possibility of performing several collections per day. Nonetheless, its instrumentation has some limitations. For example, it was necessary to adapt the duration of the saliva collection if salivation was low or if it was difficult to initiate mouth opening. Moreover, it is challenging to carry out this protocol with a single intervener, as the transport of the monitors at the same time as a patient in a wheelchair is complicated. On the other hand, it is feasible for one person alone to perform the saliva collection.

Regarding the interpretation of salivary cortisol levels in the samples and the effect of the environment on stress levels, the results should be viewed with caution and do not allow for definitive conclusions at present. Based on the analyses performed, we could not verify the hypothesis that a natural environment might reduce cortisol values. Hence, these results do not corroborate other studies of young healthy participants who participated in sessions lasting 10-30 minutes of sitting in forests compared to cities and for whom salivary cortisol levels were significantly lower in the forest groups than in the urban groups (see [20,21] for reviews). The lack of results in favor of our hypothesis on cortisol could be related to the heterogeneity of the population included in our study as well as to the pathological clinical state of the patients. In addition, the rhythm of the hospital environment that patients experience during their stay could expose them to factors influencing cortisol levels (e.g., unfamiliar environment, medical staff rotation, physiotherapy sessions, diagnostic tests and monitor noises).

The analyses performed did show a significant interaction effect between the environment and a difference in heart rate. In particular, HR varied in an opposite way to cortisol values during sessions taking place in the garden. This observation does not confirm the results of studies showing a decrease in cortisol values concomitant with vital constants in healthy participants in natural environments (garden, forest, park) or during moments of landscape observation [20,21]. In the case of our study, we can suggest that the difference found in the outdoor environment between cortisol and heart rate may be related to the difference in speed of action between the two systems involved in the stress response (autonomic nervous system (ANS) and hypothalamic-pituitary-adrenal (HPA) axis). Indeed, the ANS acts more rapidly and directly on the heart rate, whereas cortisol secretion appears a few minutes later. In this context, a new or different event could cause temporary stress and lead to discordance between the results. This is especially true during the garden sessions, as the environment is richer and more stimulating.

An interesting trend seen in our results concerns the difference in cortisol values at T30 between patient communication types. Indeed, for patients with functional and intentional communication, cortisol values tend to decrease in both environments, whereas, for patients with communication attempts, values increase significantly in the indoor environment. Studies have shown that people with aphasia have fewer coping resources and therefore experience more significant stress [22]. Comparing healthy subjects to patients with aphasia, one study showed an unusual increase in cortisol levels on awakening, and another found higher levels of salivary cortisol in the afternoon [23,24]. Despite the limited number of studies measuring cortisol in people with aphasia, the results obtained in our study support the trend of increased perceived stress in patients with severe communication impairment. Furthermore, our results could suggest that being in a closed room with few ways to interact and/or understand the situation could increase the stress level of these patients, while the same situation in an outdoor environment would act less as a stressor.

Finally, the subjective interpretation of the caregivers was in favor of an increased sense of perceived stress in the indoor environment for the patients compared to the garden session. These results corroborate numerous studies reporting increased feelings of comfort and calmness during activities taking place in outdoor settings (see [3] for a review).

Our study has a number of limitations regarding the interpretation of salivary cortisol levels. First, the small sample size, the heterogeneity of the patients included, and the limited number of salivary samples collected may have affected the power of this study. Therefore, studies with a larger population and additional salivary samples would provide a better understanding of the relationship between cortisol values in brain-injured patients and exposure to natural environments. Second, the exogenous administration of corticosteroids, which concerned 17.6% of our participants, may have limited the interpretation of the results obtained, as glucocorticoid intake may have an impact on the HPA axis. In particular, short-term treatment with high doses of glucocorticoids could lead to suppression of the HPA axis response or significantly cross-react and thus alter the secretion of endogenous cortisol. It would, therefore, be advisable in the future not to include patients treated with corticosteroids, given the difficulty of predicting the risk of interference with the HPA axis during these treatments. It would also be appropriate to control as much as possible the use of other medications that may interact with and affect the HPA axis.

Furthermore, given the unclear link between cortisol levels and TBI, it may be worth exploring other biomarkers of neuronal damage that can be measured in saliva (e.g. tau, or neurofilament heavy and light chain proteins). Finally, the use of a single salivary cortisol measurement per day does not fully reflect the diurnal cortisol cycle and baseline values for each patient. To overcome this, it would be interesting to use several salivary samples on a 24-hour cycle to know the diurnal progression of salivary cortisol levels for each patient and take into account interindividual variability. Establishing such a baseline would also allow for a more refined measurement of pituitary function and explore the possible damage of the brain injury on this function. However, this would require an increased availability of staff and organization of the service. A promising alternative for measuring physiological stress would be the continuous measurement of cortisol by a sweat sensor in the form of a patch as described in [25]. This method developed by Xsensio SA [26] is currently being validated for clinical use in the endocrinology department of the CHUV.

## Conclusions

Our results confirm that a salivary collection protocol for patients with severe brain injury in the acute phase of hospitalization is feasible and safe and allows the detection and measurement of salivary cortisol values. Salivary cortisol evaluation is therefore appropriate and innovative for this category of patients, paving the way for future research. The preliminary results of the secondary objective do not allow at this stage to reach clear conclusions concerning the influence of exposure to a natural environment on the stress level of these patients. Despite the limitations mentioned, the results are encouraging and a prospective study on a larger collective of patients would be valuable in order to evaluate the influence of natural environments on brain-injured patients and thus support the benefits of nature as an additional resource as part of their neurorehabilitation.
